# Global visual confidence

**DOI:** 10.3758/s13423-020-01869-7

**Published:** 2021-03-25

**Authors:** Alan L. F. Lee, Vincent de Gardelle, Pascal Mamassian

**Affiliations:** 1 Department of Applied Psychology, Lingnan University, Tuen Mun, Hong Kong; 2 Wofoo Joseph Lee Consulting and Counselling Psychology Research Centre, Lingnan University, Tuen Mun, Hong Kong; 3 Paris School of Economics and CNRS, Paris, France; 4 Laboratoire des Systèmes Perceptifs, Département d’études Cognitives, École Normale Supérieure, PSL University, CNRS, 75005 Paris, France

**Keywords:** Confidence, Metacognition, Integration, Perception, Vision

## Abstract

Visual confidence is the observers’ estimate of their precision in one single perceptual decision. Ultimately, however, observers often need to judge their confidence over a task in general rather than merely on one single decision. Here, we measured the global confidence acquired across multiple perceptual decisions. Participants performed a dual task on two series of oriented stimuli. The perceptual task was an orientation-discrimination judgment. The metacognitive task was a global confidence judgment: observers chose the series for which they felt they had performed better in the perceptual task. We found that choice accuracy in global confidence judgments improved as the number of items in the series increased, regardless of whether the global confidence judgment was made before (prospective) or after (retrospective) the perceptual decisions. This result is evidence that global confidence judgment was based on an integration of confidence information across multiple perceptual decisions rather than on a single one. Furthermore, we found a tendency for global confidence choices to be influenced by response times, and more so for recent perceptual decisions than earlier ones in the series of stimuli. Using model comparison, we found that global confidence is well described as a combination of noisy estimates of sensory evidence and position-weighted response-time evidence. In summary, humans can integrate information across multiple decisions to estimate global confidence, but this integration is not optimal, in particular because of biases in the use of response-time information.

**Supplementary Information:**

The online version contains supplementary material available at 10.3758/s13423-020-01869-7.

## Introduction

Perception is often presented as an inference problem involving noisy inputs and uncertainty (Mamassian, 2016). How do humans acknowledge and evaluate this perceptual uncertainty? Since Peirce and Jastrow’s (1885) seminal work, researchers have addressed this question by having observers make a confidence judgment about the perceptual decision on a sensory stimulus. Studies on perceptual metacognition have detailed the link between one perceptual decision and its associated confidence (e.g., Kepecs et al., 2008; Kiani & Shadlen, 2009; Maniscalco & Lau, 2016; Zylberberg et al., 2012), offering new theoretical frameworks to study confidence (Fleming & Daw, 2017; Pouget et al., 2016). In everyday life, the confidence in a single perceptual decision can be important in many situations, for example, in tasks that require an observer to decide whether to opt out. However, we often repeat perceptual decisions over multiple stimuli rather than a single one. For instance, a radiologist may inspect tens of mammograms a day for potential tumors. A consumer may open the egg box to confirm that no eggs are cracked before buying them. When asked about one’s own ability to perform such tasks, developing a global sense of confidence for doing the task correctly could be more relevant and important than the confidence for each individual perceptual decision. Such a global confidence will be useful to predict future performance and to decide whether or not one should engage in a task (e.g., Aguilar-Lleyda et al., 2020; Carlebach & Yeung, 2020).

So far, little is known about whether and how our metacognitive system forms a general evaluation of our own perceptual performance over a set of trials. One recent study suggests that such global confidence judgments are affected by the local confidence estimates that follow each perceptual trial and by the presence of feedback (Rouault et al., 2019). However, in the absence of feedback, global confidence judgments did not really accumulate information over trials within each set. Along a similar idea, previous studies measured participants’ performance in reaching a single perceptual decision from multiple, sometimes inconsistent, sensory evidence when sensory information is presented progressively (Balsdon et al., 2020; Fleming et al., 2018). However, how confidence is constructed over multiple, unrelated, perceptual decisions remains unknown.

To address the above questions, we designed a psychophysical paradigm that combines individual perceptual decisions and global confidence judgments. Observers were presented with two sets of sensory stimuli, and chose the set for which they were more confident in performing correctly a perceptual task. This forced-choice paradigm enables us to estimate observers’ metacognitive sensitivity in evaluating their own performance (Barthelmé & Mamassian, 2009; de Gardelle & Mamassian, 2014).

To evaluate the integration of confidence information, we manipulated the set size, i.e., the number of stimuli in each set. Critically, by increasing set size, we provided the metacognitive system with more information to evaluate global confidence. If the metacognitive system integrates this information across multiple stimuli to evaluate global confidence, metacognitive sensitivity should increase with set size. At the other extreme, if the metacognitive system can only rely on one sensory stimulus per set, metacognitive sensitivity should remain constant across set sizes.

We report here two experiments. In Experiment 1, participants viewed the stimuli in two sets without making any perceptual decisions, and then indicated which set they would be more confident in. We instructed participants to make global confidence judgments by asking them to choose the set that, if they had to make a perceptual decision about a randomly sampled stimulus within that set, they would be more likely to be correct than if they had chosen the other set. In Experiment 2, participants made perceptual decisions for all stimuli in both sets before indicating which set was associated with a greater global confidence. We also compared candidate models that include different cues and weighting strategies in describing the computations that support global confidence.

## Experiment 1

### Method

#### Participants

Experiment 1 involved 50 participants. The key effect of interest was a positive set-size effect on metacognitive sensitivity (see Experiment 1: Results), but we did not find any reference to a similar effect in previous studies, so we assumed the effect to be of medium size (Cohen’s *d* = 0.50). At alpha = 0.05 and power = 0.90, a one-sample, two-tailed *t-*test would require a sample size of at least 44. All observers were naive to the purpose of the experiment, and all participants had normal or correct-to-normal vision. Informed consent was obtained from all participants. All experimental procedures were in compliance with the Declaration of Helsinki.

Experiment 1 consisted of three sub-experiments, namely sub-experiments 1A (n=15), 1B (n=13), and 1C (n=22). The design, stimulus, and procedure were identical across the three sub-experiments, except for the details described specifically below.

#### Stimulus and apparatus

The stimulus was a Gabor patch, generated by overlaying a 2D Gaussian window (radius = 1.25°, standard deviation = 0.4°) on a sine-wave grating (spatial frequency = 2 cycles/°), with Michelson contrast = 0.4. Each set was made of several Gabor patches, oriented around a fixed reference orientation tilted 30° from vertical (with a 5° jitter across sets). This reference was indicated by a red-blue arc, with the blue (or respectively red) color on the counterclockwise (or respectively clockwise) side of the reference, each side being 40° width. The arc was 1° thick, and positioned 0.52° outside the Gabor patch.

In order to abolish interactions between consecutive stimuli, each stimulus was followed by a brief mask. The mask was generated by superimposing 256 Gabor patches of identical size and spatial frequency as the stimulus, but with randomly sampled orientations and phases, and setting the contrast of the resulting image to 0.4. We generated 32 mask images and randomly selected one for each stimulus. Stimuli and masks were faded in and out, respectively, by ramping the contrast linearly from zero to 0.4 in the first 100 ms of presentation and from 0.4 to zero in the last 100 ms.

The experiments were conducted in a dim room. Stimuli were presented on a 19-in., 1,600 × 1,200 Sony CRT monitor (Experiment 1A), or a 24-in., 1,920 × 1,080 BenQ LCD monitor (Experiments 1B and 1C), with a 100-Hz refresh rate. Viewing distance was kept constant at 57 cm (for a pixel size of about 0.03° of visual angle), stabilized using a chin and forehead rest. Monitors were calibrated with a photometer and gamma-corrected, so that luminance values were linearized to a programmable range of 0–255, which corresponded to about 0–100 cd/m^2^.

#### Procedure of initial calibration

Before the main experiment, each observer went through an initial calibration phase. Observers completed an orientation-discrimination task, which would be used in the main experiment. In each trial, the Gabor patch was presented for 500 ms, followed by a mask of 300 ms. Observers judged whether the orientation of the Gabor patch pointed to the blue (i.e., counterclockwise) or the red (i.e., clockwise) regions in the reference arc. They responded by pressing the left (counterclockwise) or the right (clockwise) arrow key on the keyboard. Feedback on the accuracy was given after each response.

Each observer completed four blocks of calibration, with two blocks for each reference (in the order of either [30°, -30°, -30°, 30°] or [-30°, 30°, 30°, -30°]). In each block, we used adaptive staircases (i.e., accelerated stochastic approximation; Kesten, 1958) to control performance via *u* , the difference between the Gabor and reference orientations. Four independent staircases were interleaved across the calibration trials, with two staircases converging at 25% and two at 75% of “away-from-vertical” responses (i.e., “clockwise” responses when the reference was clockwise relative to vertical, and “counterclockwise” responses when the reference was counterclockwise to the vertical). Each staircase was terminated after the 50th trial, or when the change in |*u*| was smaller than 0.5°, whichever came earlier. To avoid early trials with unstable learning effects, we only used the data from the last two blocks (about 320 trials per observer). We fitted to these data a cumulative normal distribution function with two parameters (the mean corresponding to the point of subjective equality and the standard deviation corresponding to the reciprocal of perceptual sensitivity, or 1/sensitivity in short). From this fitted psychometric curve, we selected different stimuli (i.e., different values of *u* ) to target the same performance levels across observers.

#### Procedure of main experiment

**Fig. 1:**
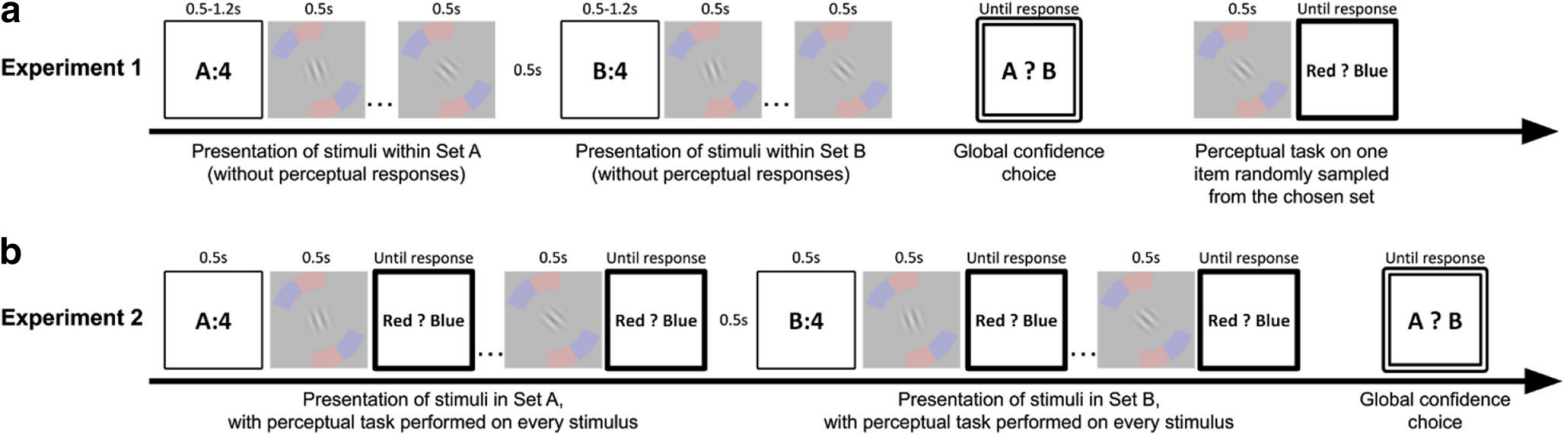
Illustration of the trial procedures for (**A**) Experiment 1 and (**B**) Experiment 2. (**A**) In Experiment 1, in each confidence-comparison trial, two sets of one, two, four, or eight oriented Gabor stimuli were presented in succession (a set size of four is shown above as an example). During the presentation of stimuli in each set, observers viewed the stimuli without giving any perceptual responses. Based on the confidence-choice response, one stimulus randomly sampled from the chosen set would be presented afterwards. Observers then performed the perceptual task on this stimulus. (**B**) In Experiment 2, procedure was identical to that in Experiment 1, except that observers performed the orientation-discrimination task on every stimulus within a set immediately after it had been presented. After the presentation of all stimuli for both sets, participants completed the global confidence-comparison task by indicating the set in which they had greater confidence overall in performing the perceptual task

Figure 1A illustrates the procedure of a trial in the main experiment in Experiment 1. In each trial, we presented two sets of stimuli, namely, set A (the first set) and set B (the second set), one after another, followed by a confidence-comparison task, and, finally, ended with one trial of the orientation-discrimination task. At the beginning of a trial, the observer saw a prompt, for example, “A:4”, which referred to the set label (A) and the set size (4, i.e., the number of stimuli to appear). This prompt lasted for 1,200 ms in Experiment 1A, and 500 ms in Experiments 1B and 1C. Then, a series of stimulus-mask pairs were presented back-to-back, with each stimulus being presented for 500 ms, followed by a mask for 300 ms. After the predetermined number of stimulus-mask pairs had been presented for set A, there was a 500-ms rest interval. Then, the prompt for set B was presented (e.g., “B:4”), followed by the presentation of stimuli in set B, which were presented in the same manner as their counterparts were presented in set A.

Set A and set B always had the same set size (e.g., in Fig. 1A both had four stimuli), but set sizes were randomized and counterbalanced across trials. The two sets were also assigned opposite reference angles: counter-clockwise for A and clockwise for B, or vice versa (in a randomized and counterbalanced order). Within each set, the reference orientation remained the same, and, therefore, the same red-blue reference arc stayed on the screen until the end of stimulus-mask series. During the presentation of stimuli in the two sets, observers were instructed not to give any explicit response to the perceptual task, but to pay attention to the stimuli within a set.

After the presentation of stimuli in both sets, observers completed a two-interval, forced-choice (2IFC) task of confidence comparison: they were asked to choose, between set A and set B, the set with stimuli on which they were more confident in performing the orientation-discrimination task. Observers responded by pressing either “1” (for set A) or “2” (for set B) on the computer keyboard. Immediately after the response, observers completed one single trial of the orientation-discrimination task. The stimulus for this trial was randomly selected from the set that had just been chosen to be “more confident” by the observer. No feedback was given to the confidence-comparison task or the one trial of orientation-discrimination task. Each observer completed eight blocks of 32 trials, resulting in 64 confidence-comparison responses for each of the four set sizes. After each block, overall accuracy on the orientation-discrimination task was given (as a percentage score) to the observer as a block feedback.

There was a targeted performance level for each of the two sets within each trial. Individual performance levels of the stimuli within each set were determined by random sampling around the targeted performance level of the set. With the sampled performance level for each stimulus, we referred to the observer’s psychometric function estimated based on the calibration data to obtain the actual *u* value for the stimulus. Here we report the targeted difficulty levels in terms of d’ for the orientation-discrimination task.

Stimulus difficulty was determined based on the psychometric curve estimated from the responses in the initial calibration phase. Each stimulus had a targeted difficulty in *d’* units, which was sampled from a normal distribution over *N*(μ,σ^2^). In Experiment 1A, the two sets were assigned with a fixed sampling standard deviation of σ=0.5, but a sampling mean of either μ=1.5 or μ=1, which correspond to 77% or 69% accuracy, respectively, in a one-interval, two-choice discrimination task for an unbiased observer. This resulted in four types of confidence-comparison trials, namely, μ_A_=μ_B_=1 for both sets, μ_A_=μ_B_=1.5 for both sets, μ_A_=1 and μ_B_=1.5, and μ_A_=1.5 and μ_B_=1, with 32 trials for each type. When the two sets had the same sampling mean difficulty level, there was no objectively “correct” answer to the 2IFC confidence-comparison task. Therefore, we focused on the trials in which sets A and B had different sampling mean difficulty. For each set size, there were 64 trials in which the sampling mean d’ values were different between the two sets.

In each of Experiments 1B (N=13) and 1C (N=22), set A and set B always had different sampling mean d’ values across all 64 confidence-comparison trials, in which half (32 trials) assigned a higher target d’ for set A. To make the confidence-comparison task easier, we increased the difference in sampling mean d’ values between the two sets and reduced the sampling variance. In Experiment 1B, we used *N*(0.71, 0.32) or *N*(1.52, 0.32), in which the mean *d’* corresponded to 64% and 78% accuracy in a one-interval, two-choice discrimination task for an unbiased observer. In Experiment 1C, we used *N*(0.57, 0.41) and *N*(1.81, 0.41) (with corresponding accuracy levels of 61% and 82% for an unbiased observer).

### Experiment 1: Results

**Fig. 2:**
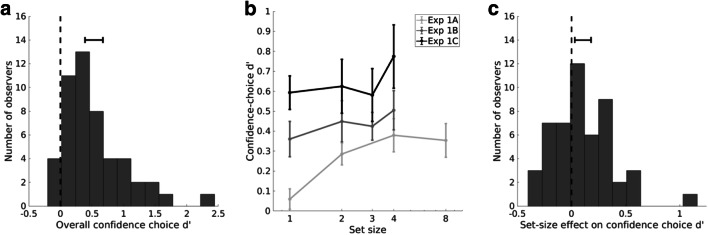
Results of Experiment 1. (**A**) Histogram of overall confidence-choice d’ across observers. Solid horizontal line with notches shows the 95% confidence interval for the mean. (**B**) Change in confidence-choice d’ as a function of set size across Experiments 1A, 1B, and 1C. Error bars represent ± 1 standard error of the mean. (**C**) Histogram of set-size effects across observers. Solid horizontal line with notches shows the 95% confidence interval for the mean

We defined a confidence choice as being correct if the participant chose the set containing easier trials (i.e., the set with the higher targeted perceptual performance). To quantify metacognitive sensitivity in the confidence-choice task, we computed the d’ (for a 2IFC task) by measuring hits (and respectively false alarms) as being confident on the first interval when that interval did contain (and respectively did not contain) the set of easier trials. Overall, the confidence choice d’ was significantly above zero (Fig. 2A, *M* = 0.531, *SD* = 0.498, 95% confidence interval (CI) = [0.390, 0.673]; one-sample t-test against zero: *t*(49) = 7.539, *p* = 1e-9, Cohen’s *d* = 1.066), indicating that participants could reliably identify the set containing easier trials.

#### Set-size effect on metacognitive sensitivity

In general, confidence choice d’ increased as set size increased (Fig. 2B). To quantify the effects for each observer, we defined the “set-size effect” as the change in metacognitive sensitivity over set sizes. For each observer, we measured the set-size effect by computing the simple linear regression slope of metacognitive sensitivity (measured in d’ units) against set size (in natural-log units). A positive slope represents an increase in metacognitive sensitivity with set size, as one would expect if observers were able to integrate metacognitive signals over multiple perceptual decisions.

Out of all 50 observers, the set-size effect was positive in 32 observers. The average set-size effect was significantly different from zero (Fig. 2C, *M* = 0.106, *SD* = 0.264, 95% CI = [0.111, 0.688]; *t*(49) = 2.839, *p* = 0.007, Cohen’s *d* = 0.40, Bayes Factor = 5.399, favoring the alternative). This result still holds even after removing one potential outlier on the positive side (set-size effect = 1.0504, *Z* score > 3.58; see Online Supplementary Material for the statistics). Although set-size effects of Experiments 1B and 1C appeared to be smaller than that of Experiment 1A, which could be related to the difference in set sizes used in respective experiments, we found that set-size effects did not differ across Experiments 1A, 1B, and 1C (one-way ANOVA, *F*(2, 47) = 0.178, *p* = 0.87; Bayes Factor = 0.177, favoring the null). We have performed further analyses to verify that the set-size effect was not correlated with local metacognitive sensitivity (when set size = 1; see Online Supplementary Materials, Fig. S1).

In summary, in this first Experiment, we found that observers were able to reliably choose the set that contained easier trials. Most importantly, the performance in this global-confidence, forced-choice task improved as set size increased, suggesting observers integrated confidence information over multiple stimuli.

However, because observers made the confidence choice *before* making a perceptual decision in Experiment 1, we could not assess how individual stimuli within the sets might have influenced the global confidence choice. In Experiment 2, we instructed observers to make the confidence choice *after* they had made a perceptual decision on every stimulus in both sets. This would allow us to measure perceptual performance across multiple decisions and examine the relationship between individual perceptual decisions and the global confidence choice.

## Experiment 2

### Method

The general method, including the stimuli, apparatus, perceptual task, and global-confidence task were the same as Experiment 1, except for the following.

#### Participants

Twenty observers participated in the experiment. Based on Experiment 1A, which used the same set sizes [1, 2, 4, 8] as Experiment 2 (see Procedure of main experiment below), the effect size for the set-size effect in Experiment 1A was *d* = 0.757. So, at power = 0.90, alpha = 0.05, a one-sample, one-tailed t-test requires a sample size of at least 17. All observers were naive to the purpose of the experiment, and had normal or correct-to-normal vision when they participated in the experiments. Informed consent was obtained from all participants. All experimental procedures were in compliance with the Declaration of Helsinki.

#### Procedure of initial calibration

Before the main experiment, each observer went through the same initial calibration phase with the identical procedure to that in Experiment 1, except that there were exactly 60 calibration trials in each of the four calibration blocks (240 trials in total), so that none of the four interleaved staircases terminated before the end of the calibration.

#### Procedure of main experiment

Figure 1B illustrates the procedure of a sample confidence-comparison trial in Experiment 2. In each trial, we presented two sets of stimuli, first set A and then set B. Each set was preceded by a prompt for 500 ms, which indicated the set label and the set size (e.g., “A:4”), i.e., the number of stimuli to appear task on this stimulus. The response keypress triggered the presentation of the second stimulus, and the procedure repeated until the participant had performed the orientation-discrimination task on all stimuli within the set. After Set A, the same procedure repeated for the presentation and orientation-discrimination task for the stimuli in Set B. There was a 500-ms interval between the two sets.

After participants had viewed and completed the orientation-discrimination task on all stimuli in both sets, they were instructed to choose the set with stimuli on which they were more confident in being able to correctly perform the orientation-discrimination task, by pressing either “1” (for set A) or “2” (for set B) on the computer keyboard.

Participants completed the whole experiment in two separate sessions (maximum gap = 8 days), with a calibration session followed by seven blocks of experimental trials (with 32 confidence-comparison trials per block) in each session. The four set sizes (1, 2, 4, and 8) were randomly interleaved within each block. In total, each participant made 448 confidence comparisons (i.e., 112 comparisons on each set size) over 3,360 perceptual decisions.

Stimulus difficulty of the orientation-discrimination task for the first block of Experiment 2 was calibrated based on the psychometric curve fitted from the initial calibration session. Starting from the second block of Experiment 2, stimulus difficulty was calibrated based on the expected performance computed from the preceding 480 orientation-discrimination trials. This allowed the calibration to closely follow changes in perceptual sensitivity across responses (corresponding to d’ levels of 1.0 and 2.0). The two sets always had different target accuracy levels. The target accuracy for individual stimuli within the set was then defined as p=0.5+q/2, where q was independently sampled from a beta distribution: *Beta*(10q, 10(1-q)). This procedure produces sampling distributions resembling two normal distributions *N*(1.0, 0.48) and *N*(2.0, 0.63) on d’, while avoiding negative d’ values.

#### Model comparison

To further investigate the computational processes underlying confidence integration, we conducted two model-comparison analyses. In the first analysis, we sought to identify the best summary statistics employed by observers for confidence integration. In the second analysis, we evaluated the position-specific weight strategy for integrating individual perceptual decisions for making a global-confidence choice.

We used the same logistic-regression framework for both analyses. Under this framework, we considered two cues for individual perceptual decisions that an observer could potentially use for making a global-confidence choice. The first cue is an internal confidence estimate for each perceptual decision, based on a standardized measure of stimulus strength. We standardized stimulus strength based on individual perceptual sensitivity and bias, such that the mean strength of the sensory sample was set equal to a standardized form of the physical orientation difference of the stimulus relative to the reference (see Equation 4 in Online Supplementary Material for details about the standardization). Because this cue is an estimate based on the distance between the stimulus and the decision criterion (in units of d’), we denote it as DIST.

The second cue is the response speed for each perceptual decision. We used the reciprocal of the response time for each perceptual decision and denote this cue as RT. For each cue, we computed the summary statistic across all perceptual decisions within each set, and then took the difference between two sets as a predictor of global confidence choices in a logistic regression. We considered the following three regression models: DIST-only, RT-only, and DIST-and-RT. For the details of model formulation, see Online Supplementary Material.

For the first analysis on summary statistics, we compared three different summary statistic strategies, namely, minimum, simple average, and maximum. The minimum and maximum strategies refer, respectively, to taking the minimum and maximum value across decisions within a set as the set’s statistic. The simple-average strategy refers to taking the arithmetic mean across the values within the set as the set’s statistic. Combined with the three types of models (DIST-only, RT-only, DIST-and-RT), this leads to nine regressions.

For the second analysis, we considered six models, which varied in terms of which cue to include (DIST only, RT only, or both DIST and RT) and the position-specific weights (uniform or exponential weights) on the values from individual perceptual decisions. Every model contained the intercept parameter in the logistic regression. Details for the formulation of the weighting profile are presented in the Online Supplementary Material.

For each model, we found the best-fitted parameters by maximum-likelihood estimation. For each observer, we computed the Bayesian Information Criterion (BIC) for each of the six models as an approximation of –2 × log(model evidence). We then computed the overall evidence for each model by taking the average model evidence across observers. Finally, we derived the Bayes Factor (as a ratio of model evidence between two models) to compare the best model (the one with the highest averaged model evidence) with all other models.

### Experiment 2: Results

#### Set-size effect on metacognitive sensitivity

**Fig. 3:**
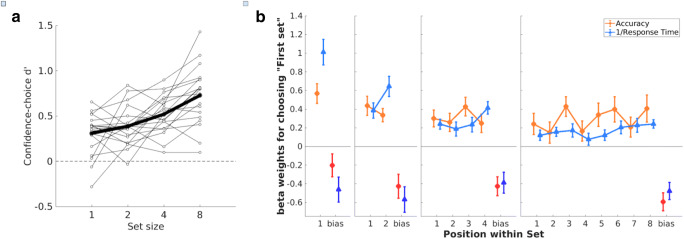
Results of Experiment 2. (**A**) Changes in metacognitive sensitivities with set size. Global metacognitive sensitivity as a function of set size for participants. Open circles (light lines) represent results of individual participants, filled circles (dark line) represent the means. The set-size effect is the slope of the regression of metacognitive sensitivity against set size (in log-units). (**B**) Weights for each position within a set to favor the first set. Weights were extracted from logistic regression analyses predicting the probability of confidence choice based on perceptual accuracy (in orange) and the reciprocal of response time (in blue) for each set size (1, 2, 4, and 8). Weights for the overall bias to choose systematically the first set are small and are shown in darker colors to the right of each panel. Error bars represent 1 SEM

Confidence-choice d’ increased as set size increased (Fig. 3A). Using the same definition for the set-size effect as in Experiment 1, we found that 19 out of 20 observers had a positive set-size effect, with the average significantly different from zero (*M* = 0.200, *SD* = 0.132, 95% CI = [0.138, 0.262]; *t*(19) = 6.787, *p* = 2 × 10^-6^, Cohen’s *d* = 1.518, Bayes Factor = 1 × 10^5^, favoring the alternative).

#### Retrospective versus prospective global confidence

Comparing the set-size effects between Experiments 1 (prospective global confidence) and 2 (retrospective global confidence), we found that set-size effect was significantly higher when observers made the global-confidence choice retrospectively than prospectively (because of the unequal sample sizes and variances, we used the independent-samples Welch’s t-test, *t*(59.004) = 2.579, *p* = .012, Cohen’s *d* = 0.608; Bayes Factor = 1.585, slightly favoring the alternative). Nevertheless, overall confidence choice d’ between the two experiments appeared to be similar (Experiment 2: *M* = 0.505, *SD* = 0.259, Welch’s t-test, *t*(56.180) = -0.152, *p* = .778; Bayes Factor = 0.270, moderately favoring the null).

#### Position-specific weighting during confidence integration

In Experiment 2, each element within a set carried the same information, on average, for the observer to make the global confidence choice. Therefore, if the observer weighted the elements optimally, global confidence should not depend on the serial position of elements within a set.

To test this hypothesis, for each observer, we separately fitted two logistic regression models, one using actual accuracy of each perceptual decision to predict confidence choice, and the other using the reciprocal of response time (1/RT; Noorani & Carpenter, 2016) for each perceptual decision to predict confidence choice. In each logistic regression, we set the position-specific evidence to be the independent variables, so that each regression coefficient β was fitted to the evidence computed at each serial position within the set. This would allow us to use the regression coefficients to quantify the weights that the observer placed on specific positions within the set. Detailed formulation of the logistic regression model can be found in the Online Supplementary Material.

We chose accuracy and RT because they are related to perceptual confidence. For accuracy, it is directly related to the global-confidence choice task, because observers were asked to choose the set in which their perceptual-task performance was better (i.e., their accuracy was higher). For RT, previous studies have shown that it is related to confidence judgments (e.g., Kiani et al., 2014).

Figure 3B shows the regression coefficients for each position, separately for analyses based on accuracy (in orange) and on response times (1/RTs, in light blue). Coefficients were positive for the accuracy analysis, indicating metacognitive sensitivity (i.e., participants chose the set with higher perceptual accuracy). In addition, as expected from studies showing that response times are a good proxy for confidence (Kiani, Corthell, & Shadlen, 2014), coefficients were also positive for the analysis based on 1/RT (i.e., participants chose the set with shorter RTs).

For an ideal confidence integrator, all weights should be equal for a given set size, but for human observers, a large magnitude of β_*i*_ weight indicates that position *i* was contributing heavily to their inference of global confidence. In Fig. 3B, the accuracy-based analysis (orange lines) shows that no specific positions were weighted more heavily than others. However, the RTs-based analysis (blue lines) shows that observers assigned heavier weights to later positions within a set for set sizes of 2 and larger. We conducted further analyses and found that the weights were constant across set positions for accuracy (all *p*s > 0.392, all Cohen’s *d*s < 0.20; all Bayes Factors < 0.327, favoring the null) but heavier in later positions for RT (all *p*s < 0.05, all Cohen’s *d*s > 0.47, all Bayes Factors > 1, favoring the alternative). For detailed statistics and an additional analysis to rule out the possibility that such RT recency effect is due to a generic response pattern, see the Online Supplementary Material.

In summary, we found that global confidence judgment was more influenced by the more recent RTs than by the earlier ones. It should be clarified that, in the above position analyses, we aimed at identifying potential statistical relationships between serial positions of elements and global confidence choices across trials. Our goal was not to assess whether observers actually used accuracy or RT information in making global-confidence choices. In order to examine the confidence integration process, for example, assessing whether position-weighted RTs of individual decisions could explain global confidence choices, we conducted the following model-comparison analyses.

#### Model comparison

To examine further the computation underlying global confidence across a set of perceptual trials, we compared three possible summary statistics (minimum, simple average, and maximum) that could be used by observers. Using a logistic-regression approach, we found that confidence choices were better predicted by a simple averaging strategy than a maximum or a minimum strategy over the set (Table 1). This was true irrespective of whether global confidence was predicted on the basis of stimulus strength (the DIST-only model), response-speed (the RT model), or both (the DIST-and-RT model).

**Table 1:** Log-likelihoods of the three summary statistics for each model, averaged across observers

Log-likelihoods averaged across observers (n=20)				
		Summary statistics		
		Minimum	Simple average	Maximum
Models	DIST-only	-276.38 (1/20)	-268.02 (*17/20)	-273.47 (2/20)
	RT-only	-271.32 (5/20)	-268.38 (*14/20)	-283.59 (1/20)
	DIST-and-RT	-258.78 (3/20)	-250.88 (*16/20)	-264.77 (1/20)

Parentheses indicate the number of observers (out of 20 for each model) with the specific summary statistic yielding the maximum likelihood. Asterisks indicate the above-chance proportions among all 20 observers. Regardless of the model, simple average provides the best fit among the three summary statistics across observers

The proportion of observers with the simple-average strategy yielding the maximum likelihood was significantly above chance (1/3 or 0.33) for all models. This suggests that the summary statistics that observers used for global-confidence integration was best described as simple averaging across multiple perceptual decisions.

Here, we did not consider summation as a possible summary statistic, which could produce different results than the simple average when set sizes differed between the two comparison sets. As we matched set size between the two comparison sets in the present study, summation and simple average would produce the same results. Future studies can explore the difference between averaging and summation by presenting different set sizes in the two comparison sets.

In a subsequent analysis, we considered how observers might put different weights on the different trials as a function of their temporal positions in the sequence. We found that the model evidence (ME) was the highest for a model featuring uniform weights on DIST and exponential weights on RT (Model 5; see Table 2). All Bayes Factors comparing Model 5 against the other models (BF_5i_ = ME_5_ / ME_i_) provide moderate or strong evidence in favor of Model 5, except for the one against Model 3, which only provides weak evidence in favor of Model 5. See Online Supplementary Material for more detailed comparison.

**Table 2:** Results of comparing models with different cues and position weights. The model featuring uniform weights on DIST and exponential weights on RT (Model 5) has the highest model evidence among all models. The Bayes Factors (BF_5i_) are all in favor of Model 5 (i.e., BF_5i_ > 1 for i = 1, 2, 3, 4, and 6)

Model	DIST Factor	RT factor	log(Model evidence) averaged across observers	BF_5i_ (Bayes Factors for comparing the best model (Model 5) against Model i)
1	uniform	absent	-274.12	2.36e+6
2	absent	uniform	-274.48	3.38e+6
3	uniform	uniform	-260.04	1.80
4	exponential	uniform	-262.16	15.01
5	**uniform**	**exponential**	**-259.45**	**1**
6	exponential	exponential	-261.70	9.48

For the best-fit model, the temporal weight parameter was above zero on average (*M* = 1.272, *SD* = 3.941, 95% CI = [-0.572, 3.117]), suggesting that observers could have weighted later items heavier in general. Because the fitted parameter for one observer appeared to be an outlier (over 4 standard deviations above the mean; see Online Supplementary Material, Fig. S3, for the full distribution of the fitted parameter values), we performed the nonparametric Wilcoxon signed-rank test and found that the median was significantly above zero (*Z* = 2.0533, signed-rank sum = 160, two-tailed *p* = 0.04).

Overall, the results from model comparison suggest that the integration of confidence over multiple perceptual decisions is best described with a model that includes two cues: one as the average across local confidence estimates, the other as the position-weighted promptness for individual decisions.

## Discussion

Recent studies have suggested that confidence regarding our current decision may be estimated by carrying over information from past trials in the task (e.g., Aguilar-Lleyda et al., 2021; Meyniel et al., 2015; Purcell & Kiani, 2016; Rahnev et al., 2015). These studies thus hint at the notion of a global confidence, which was inferred from the observers’ decisions using computational modelling. In the present study, we addressed this issue more directly by asking observers to make global-confidence judgments over sets of trials. By evaluating metacognitive sensitivity across set sizes, we found that observers made better global-confidence judgments when more information (i.e., more items) was available, suggesting that the metacognitive system integrates confidence information across multiple perceptual decisions. In the Online Supplementary Material, we also rule out the possibility that the set-size effect on metacognitive sensitivity would be due to the representation of confidence becoming more normal (and thus better suited to sensitivity analysis) as set size increases. Our analyses showed that although the fidelity of the normality assumption indeed improved with set size, this was not the cause of our results. Instead, the increase in metacognitive sensitivity with set size was mediated by the enhanced signal-to-noise ratio in the internal representation of global evidence (see Figs. S4–S6 and Table S2 in Online Supplementary Material).

Our results are also consistent with recent findings that information from “local” confidence is used when generating “global” estimates of performance (Rouault et al., 2019). However, Rouault et al. (2019) did not find a significant set-size effect (unless feedback was given following the local perceptual decisions). There are multiple possible explanations for this discrepancy with the present study. First, their set sizes were larger on average, creating longer continuous streaks than in the present study. Second, their easy and difficult trials were randomly interleaved. Observers could only categorize trials into the sets based on a pre-trial cue. This could create more noise in representing and storing local confidence estimates compared with the present study, in which easy and hard trials were always grouped and presented consecutively within the same set. Third, in Rouault et al., trials with feedback and without feedback were interleaved, whereas in the present study, no trial-by-trial feedback was provided. The presence of feedback could have led participants to adopt a different integration strategy over all trials, which could affect the without-feedback trials.

Our results also show that global confidence was influenced by RTs from past decisions. Specifically, our findings suggest a recency effect for RTs (i.e., greater influence from RTs in more recent decisions), and in comparison, the influence from perceptual accuracy was uniform across serial positions. This may appear to be contrary to Rouault et al.’s (2019) findings, as neither accuracy nor RT seemed to have influenced global confidence choices. However, this could be due to the fact that our analysis on accuracy and RT was specific to the position at which each item was presented in the set, and theirs was on the overall influence from average accuracy and RT. While RTs could be partially accessible to participants (e.g., Gorea et al., 2010) and are related to confidence judgments (e.g., Baranski & Petrusic, 1994; Kiani et al., 2014), the present study is, to our knowledge, the first to identify a position-specific weighting of RT information during confidence integration. If observers used RTs to estimate global confidence, this explicit use would be more heavily affected by memory limitations and could explain the RT recency effect. Nevertheless, the observation that recent RTs contributed more than early ones suggests that the confidence-integration mechanism is not optimal. The benefits, if any, of this overweighting of recent decisions for global confidence remain to be explored.

The suboptimality of global confidence integration described in the present study may require further investigation. For instance, if memory limitations could explain the RT recency effect, leading to suboptimal global confidence integration, then why would memory limitations not affect the position weights on accuracy? We did not find such a recency effect in the integration of sensory evidence either (i.e., the finding that the best model weighs DIST uniformly). This suggests that the degree of suboptimality may vary when observers integrate different types of evidence. Future studies can explore this evidence-specific suboptimality in confidence integration.

Finally, the present study demonstrates that confidence integration takes place for both prospective and retrospective global-confidence judgments. The apparently stronger set-size effect in retrospective judgments suggests that explicit perceptual decisions made *before* global confidence judgments could facilitate confidence integration. Interestingly, despite this stronger set-size effect for retrospective global confidence, the overall global-confidence sensitivity was similar between retrospective and prospective conditions. This contrasts with previous work showing that metacognitive sensitivity in an anagram task was lower when confidence ratings were given before responding to the task (Siedlecka et al., 2016). However, we acknowledge that our study was not originally designed to compare global retrospective and prospective confidence in the same participants under the same experimental conditions. Also, Experiments 1 and 2 differ in terms of many other factors. In particular, the procedure in Experiment 2 was more engaging, which could lead to better confidence integration and thus a stronger set-size effect. As the present study may not allow us to make a strong claim on this issue, future studies can systematically examine the effects of retrospective/prospective judgments on confidence integration. In general, one important avenue for future research is to clarify the relations among local, global, retrospective, and prospective confidence judgments.

## Supplementary Information

ESM 1 (PDF 416 KB)
