# Supplementary material for: Global visual confidence
Source: Psychon Bull Rev. 2021 Mar 25;28(4):1233–42. doi: 10.3758/s13423-020-01869-7 (PMC8367895; doi:10.3758/s13423-020-01869-7)
Supplement: Supplementary file 1 — (PDF 416 KB) [file 13423_2020_1869_MOESM1_ESM.pdf]

SUPPLEMENTARY MATERIALS

TEST FOR SET-SIZE EFFECT WITH OUTLIER REMOVED

The average set-size effect was still significantly different from zero even after the outlier had been removed (Figure 2C, M = 0.087, SD = 0.228, 95% C.I. = [0.0211, 0.152]; t(48) = 2.83657, p = .011, Cohen's d = .38, Bayes Factor = 3.582, favoring the alternative).

INDEPENDENCE BETWEEN THE SET-SIZE EFFECT AND LOCAL CONFIDENCE D'

We checked whether there was any relationship between the set-size effect we reported in the main text and the observer's metacognitive sensitivity. Metacognitive sensitivity was estimated when there was only one item in the set, a condition that was identical to the typical confidence forced-choice paradigm we used in the past (e.g. de Gardelle & Mamassian, 2015). We did not find any correlation between the magnitude of the set-size effect and the metacognitive sensitivity of observers (Figure S1; with all data included (n=50): r = .008, p = .958, Bayes Factor = 0.177, moderately favoring the null; with the outlier removed (n=49): r = -0.177, p = 0.223, Bayes Factor = 0.366, slightly favoring the null). We also tested whether there was any relationship between the set-size effect and perceptual sensitivity. Perceptual sensitivity was estimated as the reciprocal of the sigma parameter of the cumulative normal used to fit the orientation-discrimination psychometric function. We did not find any correlation between set-size effect and individual observer's perceptual sensitivity in our data (with all data included (n=50): r = .117, p = .419, Bayes Factor = 0.242, moderately favoring the null; with the outlier removed (n=49): r = 0.208, p = 0.151, Bayes Factor = 0.485, slightly favoring the null).

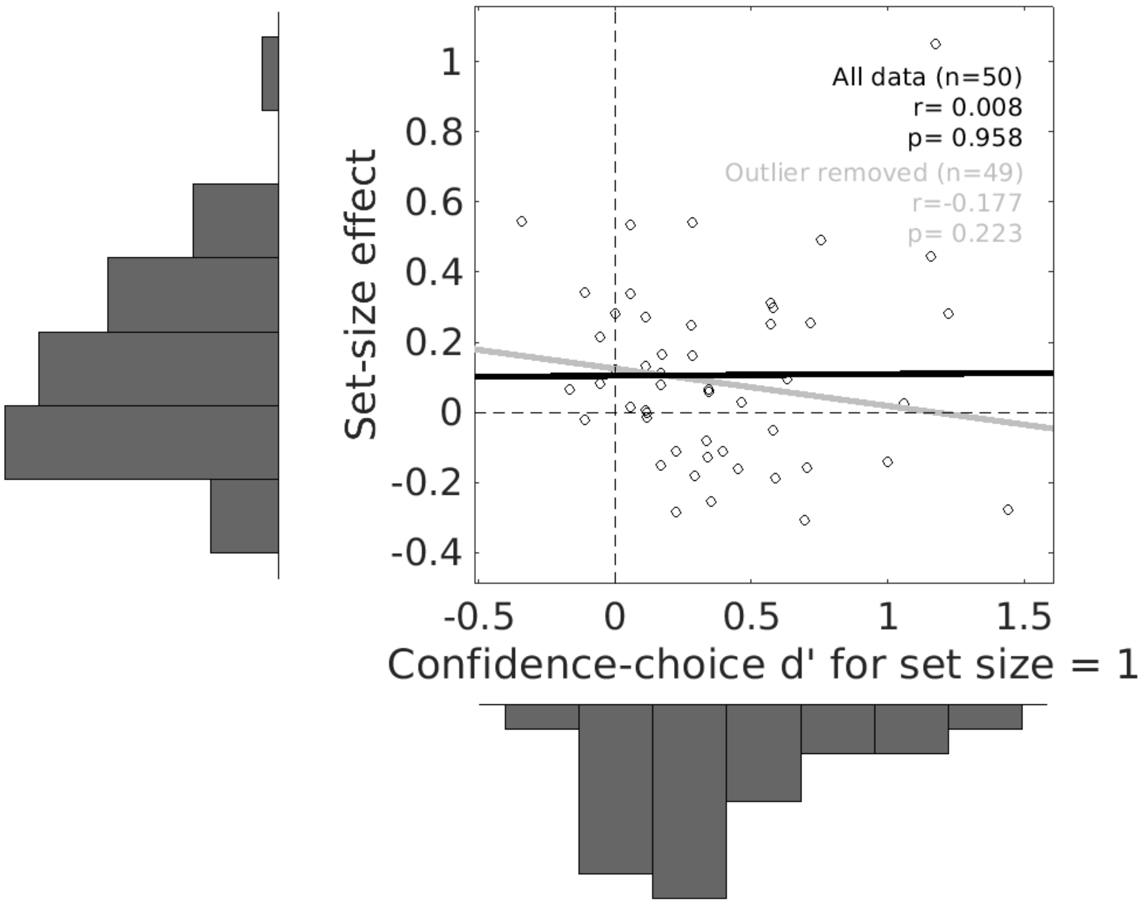

Figure S1. Correlation between set-size effect and local confidence d’ (i.e., for set size = 1) across observers. Correlation was computed both with all data (n=50, dark text and fitted line) and after removing the outlier mentioned in the main text (n=49, gray text and fitted line).

ANALYSIS ON SERIAL-POSITION EFFECTS

Logistic regression In the main text, we described a logistic-regression analysis on specific serial positions that could differentially influence global confidence choice. In these analyses, we assumed that the integration mechanism computed a linear weighted sum of estimated perceptual uncertainty for each set. Then, global confidence choices were made by comparing the two sets of uncertainties.

In the following analysis, we use position-specific perceptual accuracy and response time as two candidates for estimated perceptual uncertainty. We further assumed that the weight for the same serial position was the same between both sets. Formally, global confidence choice C is a function of θ, which represents the difference in uncertainty between the two sets, and is computed as follows

$$\theta = \sum_{i=1}^{n} \beta_i u_{A,i} - \sum_{i=1}^{n} \beta_i u_{B,i}, \tag{1}$$

where $u_{X,i}$ is the estimated perceptual uncertainty for serial position i in set X, for $X = A, B$, and β_i is the weight for serial position i for both sets. Rearranging the equation above, we have

$$\theta = \sum_{i=1}^{n} \beta_i \left(u_{A,i} - u_{B,i} \right) = \sum_{i=1}^{n} \beta_i \Delta u_i. \tag{2}$$

This allows us to compute Δu_i, the difference in estimated uncertainty at each position between the two sets, and to apply one weight value to it. To transform this difference in uncertainty into a probability of choosing one set as more confident, we used the standard logistic function

$$P\ (C = \ Set\, A) = f\,(\theta) = \frac{1}{1+e^{-\theta}}. \tag{3}$$

This logistic regression does not include any additional degree of freedom as the sensitivity to the difference in uncertainty between the two sets can directly be represented in the β_i weights.

Statistical tests for recency effects on logistic regression weights For each participant and for each set size of 2 and larger, we fitted a linear regression model to the logistic regression weights β_i over serial positions i. Table S1 shows the results for the one-sample t test against zero across subjects for both accuracy and reciprocal of response time.

**Variable**	**Set size**	**M**	**SD**	**95% C.I.**	**t (df = 19)**	**p**	**Cohen's d**	**Bayes Factor**
**Accuracy**	2	-0.100	0.509	[-0.338, 0.138]	-0.876	.392	-0.196	0.327
	4	0.001	0.157	[-0.073, 0.075]	0.031	.976	0.007	0.232
	8	0.018	0.095	[-0.026, 0.063]	0.854	.404	0.191	0.321
	avg.	-0.027	0.190	[-0.116, 0.062]	-0.630	.536	-0.141	0.278
**1/RT**	2	0.258	0.437	[0.054, 0.463]	2.642	.016	0.591	3.462
	4	0.057	0.119	[0.001, 0.113]	2.136	.046	0.478	1.486
	8	0.016	0.028	[0.003, 0.029]	2.545	.020	0.569	2.918
	avg.	0.110	0.175	[0.029, 0.192]	2.824	.011	0.631	4.787

Table S1. Statistical results of one-sample t tests against zero across subjects on the linear-regression slopes (position-specific weights over serial position) for each set size larger than two. The "avg." indicates the within-observer averages of the linear slopes

across set sizes, as an estimate for the per-observer effect regardless of set sizes. Bayes Factors below 1 favor the null hypothesis (that the mean equals zero). Bayes Factors above 1 favor the alternative hypothesis (that the mean is different from zero).

Generic response pattern as an alternative explanation We need to rule out the possibility that the RT recency effect could be due to a generic response pattern that is irrelevant to confidence choices (e.g., response times increased as serial position advanced within a set regardless of confidence choice). We thus computed average perceptual accuracy and average response time across all sets and trials set for each serial position, and found that average accuracies and response times (except for the first decision within a set) remained constant over serial positions within a set for all set sizes. The absence of “recency” pattern in these averages suggests that the recency effect in response times was not generic, but was specifically related to global confidence choices. Figure S2 shows the average position-specific accuracies and response times. Average accuracies and response times (except for the first decision within a set) remained constant over serial positions within a set for all set sizes. The slow average response times in the first decision within each set might be due to attention lapse right after the prompt before each set began. The absence of “recency” pattern in these averages suggests that the recency effect in response times was not generic, but was specifically related to global confidence choices.

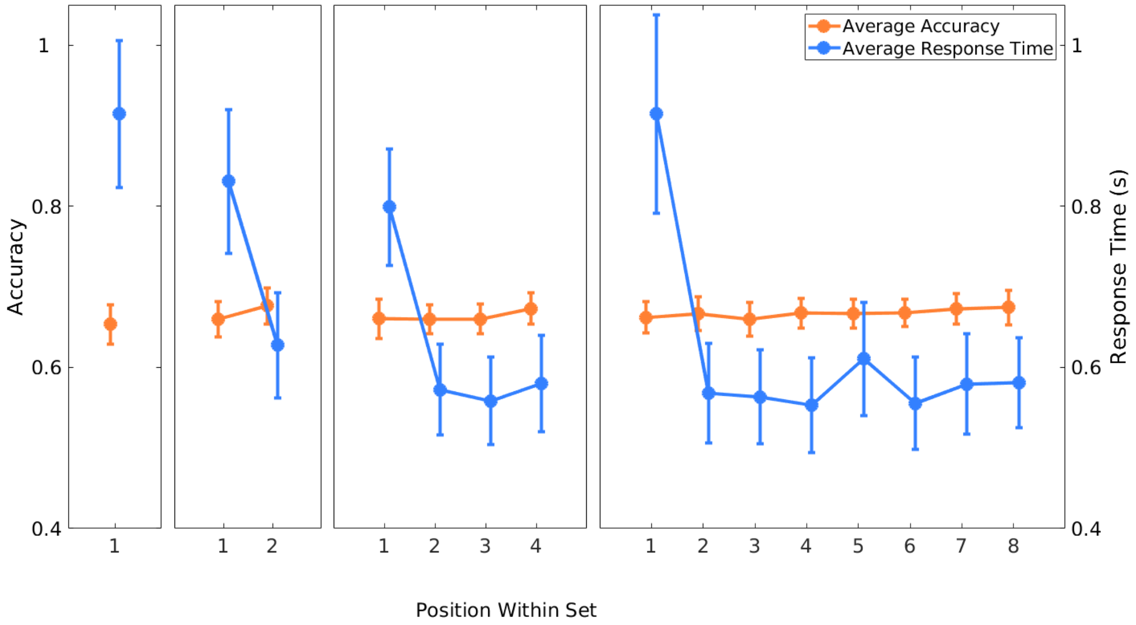

Figure S2. Perceptual accuracies (orange) and response times (blue) averaged at each serial position. Panels represent different set sizes (from left to right: set size = 1, 2, 4, and 8). Error bars represent +/- one standard error of the mean.

COMPUTATIONAL MODELS

Model formulation

We considered two cues from which an observer would gather evidence in making the global-confidence choice.

The first cue was based on the sensory stimuli within each set. In each confidence-comparison trial in Experiment 2, observers viewed two sets of Gabors, one after another. With respect to the orientation-discrimination task, the sensory information is the difference in orientation between the Gabor orientation and the reference orientation. We denote by $u_{i,j,t}$ this orientation difference for a stimulus presented at serial position i in set j in trial t with set size n, where $i \in \{1, 2, ..., n\}$ for $n \in \{1, 2, 4, 8\}$, $j \in \{A, B\}$ (the first or second set), and $t = \{1, 2, \ldots, 448\}$ (total number of confidence-comparison trials each observer completed). For each observer, we estimated the sensory noise (as σ) and perceptual criterion (as μ) by fitting the cumulative normal distribution function as the psychometric curve to all perceptual responses. Then, we standardized all sensory input $u_{i,j,t}$ by computing $Z_{i,j,t}$ as follows

$$Z_{i,j,t} = \frac{(u_{i,j,t} - \mu)}{\sigma} \quad . \tag{4}$$

This $Z_{i,j,t}$ represents the standardized "distance" between the sensory representation of the stimulus from the decision criterion. We took this as a local confidence estimate based on sensory information for each perceptual decision. We denote this cue as $DIST$.

The second cue was based on response time for each perceptual decision ($RT_{i,j,t}$) within a set. Based on similar notations as above, we computed $T_{i,j,t}$ as follows

$$T_{i,j,t} = 1 / RT_{i,j,t} \quad . \tag{5}$$

This reciprocal of the response time was taken as the local confidence estimate based on response time for each perceptual decision. We denote this cue as RT .

In the first analysis about summary statistics, we considered three ways to obtain the global confidence estimate for each set across perceptual decisions. The “average” strategy was to compute the arithmetic mean across the values within each set as the evidence estimate. For example, the $DIST$ evidence for set j in trial t, the evidence $E_{DIST,j,t}$ was computed as follows (the evidence based on response time $E_{RT,j,t}$ was computed likewise)

$$E_{DIST,j,t} = (1/n)\sum_{i=1}^{n} Z_{i,j,t} \ . \qquad (6)$$

The “maximum” and “minimum” strategies were to take, respectively, the maximum and minimum values across items within each set as the evidence estimate for the set.

In the second analysis about the position-specific weights, each local evidence estimate was weighted based on its position before being summed over. For example, for the $DIST$ cue (likewise for the RT cue)

$$E_{DIST,j,t} = \sum_{i=1}^{n} w_i Z_{i,j,t} \ . \qquad (7)$$

For uniform weights over positions, we set $w_i = 1/n$ so that the computation of evidence is identical to arithmetic mean.

For exponential weights, we computed w_i based on an exponential weight function

$$w_i = e^{(ri)} / \sum_{k=1}^{n} e^{(rk)} \ . \qquad (8)$$

The parameter r controls the variation in position weighting. When $r > 0$, later items in the set are given heavier weights, with the last item having the heaviest (i.e., simulating a "recency" effect). When $r < 0$, earlier items in the set are given heavier weights, with the first item having the heaviest (i.e., simulating a "primacy" effect). When $r = 0$, the exponential weight function is identical to uniform weights. The local estimates based on $DIST$ and RT cues could be combined using separate values of r, so that we made r_{DIST} and r_{RT} two different parameters.

Then, the evidence for set B was subtracted from that for set A to obtain the combined evidence for choosing set A to be the more-confident set for trial t. For example, for the $DIST$ evidence,

$$E_{DIST,t} = E_{DIST,A,t} - E_{DIST,B,t} \ . \quad (9)$$

The evidence based on response time $E_{RT,j,t}$ was computed likewise.

Then, the overall evidence for choosing set A was the linear combination of the evidence from the two cues, plus a constant term to capture bias in confidence choices:

$$\theta = \beta_0 + \beta_{DIST}E_{DIST} + \beta_{RT}E_{RT} \ . \quad (10)$$

The probability for choosing the Set A was modeled based on the logistic function as described in equation (3).

In terms of this model formulation, the six models differ in terms of which parameters were fitted to the data based on maximum-likelihood estimation (all models included the intercept term β_0). Below are the models we considered, with the parameters being fixed described:

Model 1: the uniform-DIST-only model (fitted β_0 and β_{DIST} only)

Model 2: the uniform-RT-only model (fitted β_0 and β_{RT} only)

Model 3: the uniform-DIST-uniform-RT model (fitted β_0 , β_{DIST} , and β_{RT} only)

Model 4: the exponential-DIST-uniform-RT model (fitted β_0 , β_{DIST} , β_{RT} , and r_{DIST} only)

Model 5: the uniform-DIST-exponential-RT model (fitted β_0 , β_{DIST} , β_{RT} , and r_{RT} only)

Model 6: the exponential-DIST-exponential-RT model (fitted β_0 , β_{DIST} , β_{RT} , r_{DIST} , and r_{RT} only)

Parameter fitting

The set of parameter(s) in each model was estimated for each observer via maximum-likelihood estimation. For each model m and observer k, the likelihood function for the parameter set $\theta_{m,k}$ was defined as the product of the probabilities that the model made the same global confidence choice as the observer did across all trials,

$$L\left(\theta_{m,k} \mid \{y_k\}\right) = \prod_t p\left(\hat{y}_{k,t} = y_{k,t}\right) \quad . \tag{11}$$

We used MATLAB’s "‘glmfit” function to obtain the logistic-regression parameters ($\beta_0,\ \beta_{DIST},\ and\ \beta_{RT}$) for a fixed set of [$r_{DIST},\ r_{RT}$], and used MATLAB’s “fmincon” function to obtain the set of [$r_{DIST},\ r_{RT}$] that maximizes the likelihood. In short, this procedure allowed us to obtain the parameter set $\theta_{m,k}$ that maximizes $L\left(\theta_{m,k} \mid \{y\}\right)$ for each observer k.

Model selection

We took the parameter set $\hat{\theta}_{m,k}$ that maximized $L_{m,k}$ as the estimated parameter set, and the associated likelihood value $\hat{L}_{m,k}$ as the maximum likelihood for model m for observer k.

We selected the best model by comparing the model evidence across models. We approximated the model evidence $ME_{m,k} = p(\{y_k\} \mid Model = m)$ for each model m for each observer k using the Bayesian information criterion BIC_m

$$log(ME_{m,k}) \approx -(1/2)\, BIC_{m,k}\,, \quad (12)$$

where

$$BIC_{m,k} = ln(T_n)\, q_m - 2ln\left(\hat{L}_{m,k}\right)\,, \quad (13)$$

T_n = 448 is the total number of global-confidence choice completed by each observer, and q_m is the number of free parameters in each model.

In the main text, we reported the computation of model evidence based on the average log model evidence across observers (N = 20 is the total number of observers), i.e.,

$$log(ME_m) = (1/N) \sum_{k=1}^{N} log(ME_{m,k})\,. \quad (14)$$

We then compared the best model (Model 5) with all other models based on the Bayes Factor

$$BF_{5m} = ME_5 \,/\, ME_m\,. \quad (15)$$

Fitted temporal-weight parameters for the best model

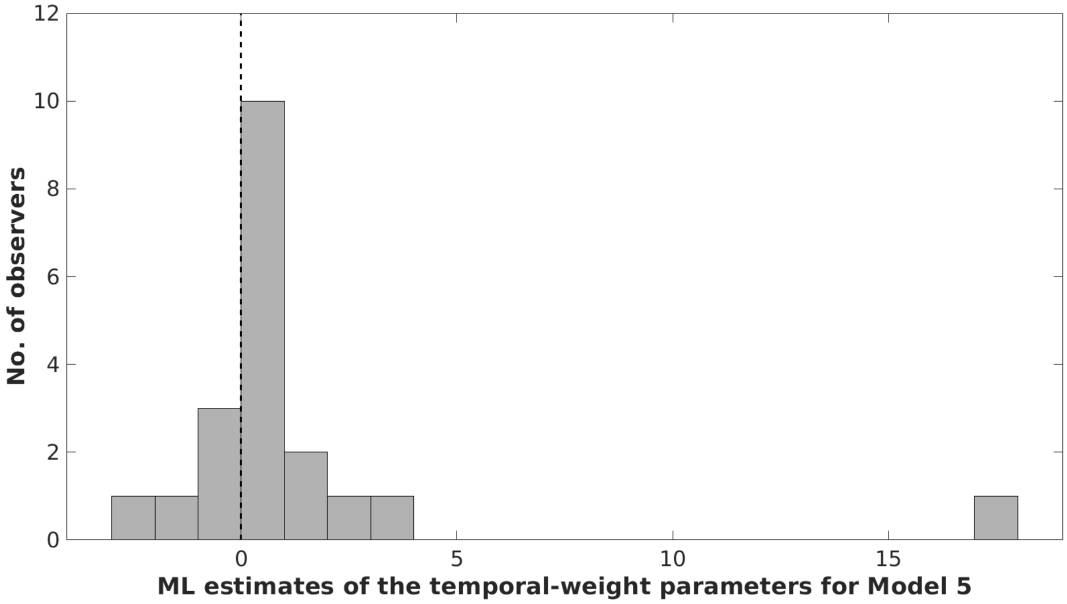

Figure S3. Histogram of the maximum-likelihood estimates of the temporal-weight parameters (r_{RT}) for Model 5 (i.e., the uniform-DIST-exponential-RT model). The fitted value for one observer (17.17) was 4 standard deviations above the mean. The vertical dashed line marks $r_{RT} = 0$.

ADDITIONAL STATISTICAL ANALYSES ON THE SET-SIZE EFFECT

Background Suppose the evidence for global confidence was computed by averaging multiple noisy local confidence estimates arising from the perceptual decisions within a set. Then, as set size increases, based on the central limit theorem, there are two consequences on the distribution of the internal representation of global evidence. First, such the global-evidence distribution becomes more similar to a normal distribution, increasing the fidelity of the normality assumption in using signal detection theory (SDT) to analyze the data. Second, the variability of the global-evidence distribution decreases for both the hard and easy sets and, with the mean being constant as set size varies, the signal-to-noise ratio for the global-confidence choice task increases.

We conducted two additional analyses on the data from Experiment 2 and found that the set-size effect on confidence-choice d’ (i.e., the increase in global metacognitive sensitivity as set size increased) was mainly due to the second consequence (i.e., increase internal signal-to-noise ratio) instead of the first one (i.e., increase in the fidelity of the normality assumption for SDT).

Simulation preparation We obtained samples of noisy local estimates by generating 100 simulated runs of Experiment 2 for each observer. In each simulation run, the noise for each local estimate was independently sampled from a Gaussian distribution, with mu and sigma, respectively, being the PSE and sensitivity of the observer’s own perceptual psychometric curve. Then, for each trial, we computed the global evidence for the easy and hard set by averaging the noisy local confidence estimates within each set. Figure S4 shows the distributions of local (top row) and global evidence (bottom row) of the two sets as set size increases.

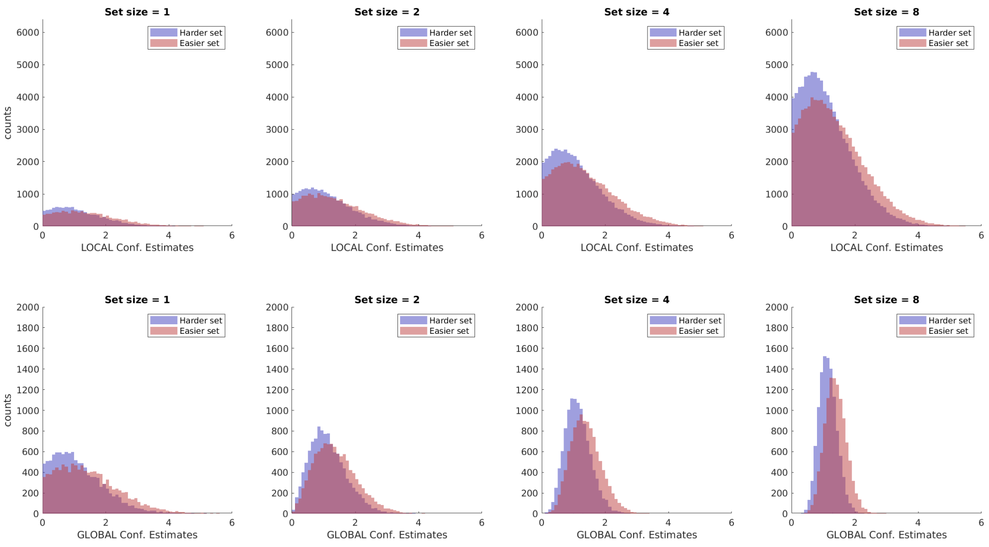

Figure S4. Distributions of local (top) and global (bottom) evidence for the easy (red) and hard (blue) sets, aggregated over the 100 simulation runs. Note that, as set size increases

(from left to right: 1, 2, 4, and 8), the global distributions become more similar to normal distributions with decreasing variances.

For each simulation run for each observer, we computed the following variables:

1. **normality of evidence**: the log-likelihood of the normal-distribution fit for the simulated global evidence (as a measure of the fidelity of the normality assumption), and

2. **internal signal-to-noise ratio (SNR)**: the difference in means divided by the pooled SD between the two sets' distributions, i.e, $SNR = (\mu_{Easy} - \mu_{Hard}) / \sqrt{s_{Easy}{}^2 + s_{Hard}{}^2}$).

3. **internal sensitivity (AUROC)**: the Area Under the Receiver Operating Characteristic curve based on the internal global-evidence distributions of the easy and hard sets.

To quantify the effects of set size on the above variables, we computed the linear slopes of regressing the respective variables by the natural log of set sizes (i.e., the same method as we computed the set-size effect for the confidence-choice d' in the main text). As a result, for each observer in each simulation run, we obtained the "set-size effect" on each of the above variables. We then averaged these set-size effects across the 100 simulation runs to obtain an estimate of each type of set-size effect for each observer.

Correlating set-size effects If any of the variables are related to the set-size effect on metacognitive sensitivity, the corresponding set-size effects should be correlated with the set-size effect on confidence-choice d'. We computed such correlation for each of the set-size effects with the set-size effect on confidence-choice d'.

Figure S5 shows the scatter plots with correlations. If the increase in fidelity of the normality assumption could explain the set-size effect on confidence-choice d', we should observe a correlation between the respective set-size effects on these two variables.

However, we did not find such a correlation (left panel below; r = .2543, p = .2793). Instead, we found a positive correlation between the respective set-size effects on confidence-choice d’ and on internal SNR (middle panel below; r = .6236, p = .0033). We found similar results between the respective set-size effects on confidence-choice d’ and on the AUROC (right panel below; r = .5762, p = .0078).

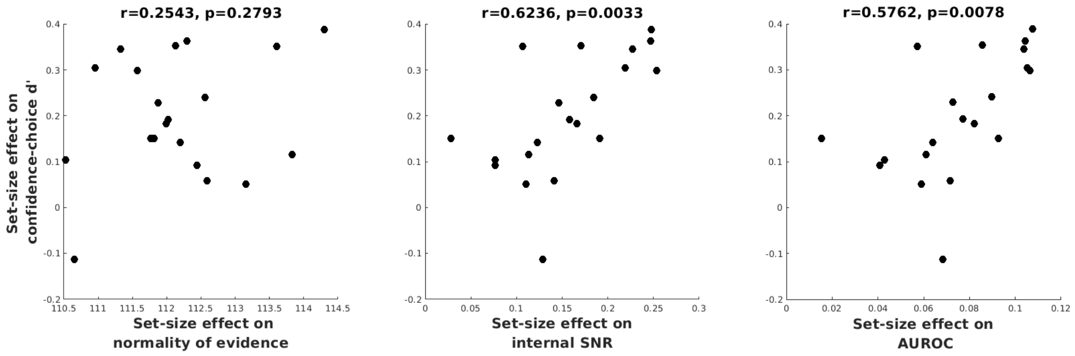

Figure S5. Scatter plots between the set-size effect on confidence-choice d’ and the set-size effects of the following variables: Left, normality of evidence (log-likelihood of fitting a normal distribution to the internal evidence distributions; averaged log-likelihood between the easy and hard sets); Middle, internal SNR of the evidence distributions of the easy and hard sets (computed as the difference in means divided by the pooled SD between the two sets’ distributions, i.e, $SNR = (\mu_{Easy} - \mu_{Hard}) / \sqrt{s_{Easy}^2 + s_{Hard}^2}$; Right: AUROC derived from the internal evidence distributions. Pearson’s correlation (r) and the corresponding p value are shown above each panel. Note that the internal SNR and AUROC are highly correlated (r = .9862, p < .0001) as both represent the separation between the distributions of global evidence of the easy and hard sets in the observer’s internal representations.

Mediation analysis To further examine the possible influence from the fidelity of normality assumption, we constructed the following mediation model. The purpose of this analysis is to evaluate the mediating effects of the following two variables.

We included four variables for each observer and each set size. For the simulated variables, we used the average across the 100 simulation runs. As a result, each variable contained 80 observations (20 observers x 4 set sizes). Figure S6 illustrates the mediation model with the fitted coefficients and statistics.

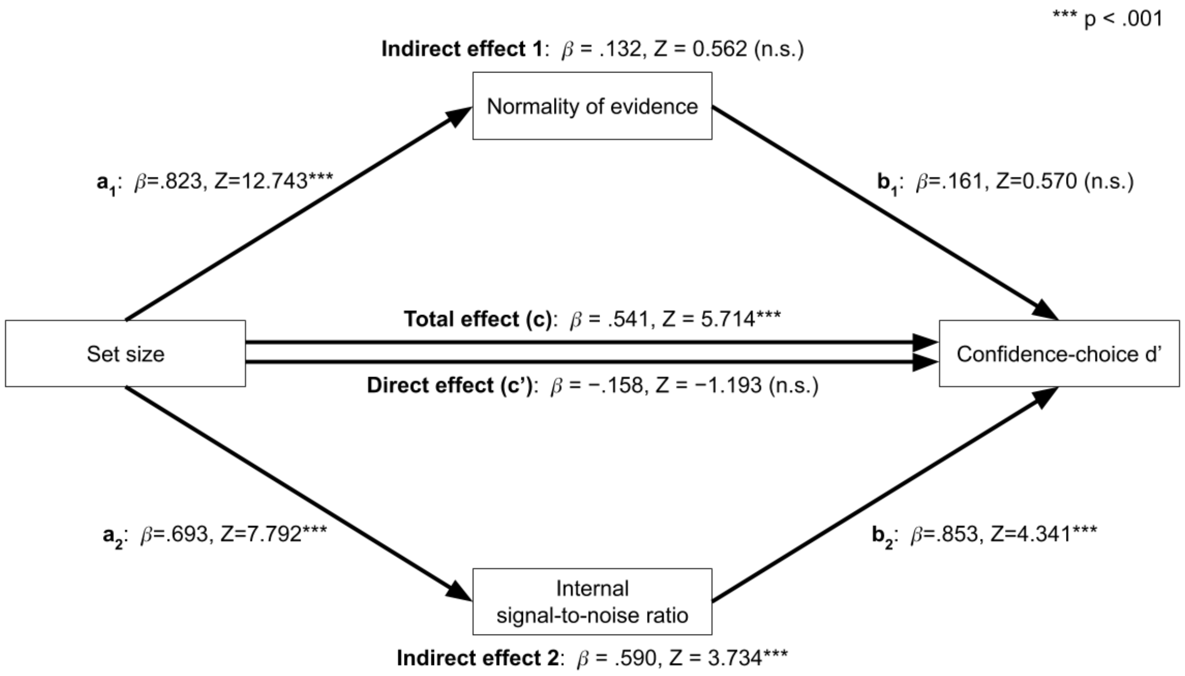

Figure S6. The mediation model. “Set size” on the left is our main IV (varied over log([1, 2, 4, 8]), and Confidence-choice d’ on the right is the d’ in confidence choice reported in the Results section of Experiment 2 in the main text. The one at the top (Normality of evidence) was the log-likelihood of the normal-distribution fit for the internal representation of global confidence in each simulation, averaged across the harder and easier sets. It represents the fidelity of the normality assumption on the representation of internal evidence. The one at the bottom (Internal signal-to-noise ratio) was the SNR in the internal

representation of global evidence. It was computed as the standardized distance between the means of the two distributions of the global evidence, one for the hard set and one for the easy sets. The β values are the standardized coefficients for each effect. The Z values are the test statistic for that coefficient (with test of significance indicated).

We estimated the coefficients of the mediation model using the Advanced Mediation Models module (Gallucci, 2019) on jamovi (The jamovi project, 2020), with confidence intervals computed based on 5000 bias-corrected bootstrap samples. Results are shown in Table S2.

Type	Effect	Estimate	SE	95% C.I. (a) Lower	Upper	β	z	p
Indirect	**1**: Set size ⇒ Normality of evidence ⇒ Confidence-choice d’	0.047	0.083	-0.120	0.192	0.132	0.562	0.574
	**2**: Set size ⇒ Internal SNR ⇒ Confidence-choice d’	0.209	0.056	0.109	0.326	0.590	3.734	< .001
Compo-nent	$\mathbf{a_1}$: Set size ⇒ Normality of evidence	112.18	8.80	94.89	129.72	0.823	12.743	< .001
	$\mathbf{b_1}$: Normality of evidence ⇒ Confidence-choice d’	4.18e-4	7.34e-4	-0.001	0.002	0.161	0.570	0.569
	$\mathbf{a_2}$: Set size ⇒ Internal SNR	0.156	0.020	0.116	0.195	0.693	7.792	< .001
	$\mathbf{b_2}$: Internal SNR ⇒ Confidence-choice d’	1.341	0.309	0.748	1.966	0.853	4.341	< .001
Direct	**c’**: Set size ⇒ Confidence-choice d’	-0.056	0.047	-0.150	0.033	-0.158	-1.193	0.233
Total	**c**: Set size ⇒ Confidence-choice d’	0.200	0.035	0.131	0.269	0.541	5.714	< .001

Table S2. Coefficients of the mediation model, with corresponding statistics and tests. Confidence intervals were computed with 5000 bias-corrected bootstrap samples. β values are completely standardized effect sizes.

We found a significant total effect of set size on confidence-choice d’ (β= .541, Z = 5.714, p < .001), which is consistent with what is reported in the main text. However, the direct effect was insignificant (β= -.158, Z = -1.193, p = .233).

Critically, we did not find any indirect effect from set size via “normality of evidence” to confidence-choice d’ was insignificant (β= .132, Z = 0.562, p = .574), which may be due to the weak path from “normality of evidence” to confidence-choice d’ (β= .161, Z = 0.570, p = . 569). Instead, we found a significant indirect effect from set size via “internal SNR” to confidence-choice d’ (β= .590, Z = 3.734, p < .001). All other component paths were significant (all ps < .001).

Taken together, these results suggest that the set-size effect on confidence-choice d' reported in the main text was not mediated by the increase in the fidelity of the normality assumption on the internal evidence distributions. Instead, a more likely mediating factor was the enhanced signal-to-noise ratio in the internal representations of global evidence.

SUPPLEMENTAL REFERENCES

de Gardelle, V., & Mamassian, P. (2015). Weighting mean and variability during confidence judgments. *PLoS ONE*, *10*(3), e0120870. http://doi.org/10.1371/journal.pone.0120870

The jamovi project (2020). *jamovi*. (Version 1.2) [Computer Software]. Retrieved from https://www.jamovi.org.

Gallucci, M. (2019). *jAMM: jamovi Advanced Mediation Models*. [jamovi module]. Retrieved from https://jamovi-amm.github.io/.
